# History and current status of polymyxin B‐immobilized fiber column for treatment of severe sepsis and septic shock

**DOI:** 10.1002/ags3.12015

**Published:** 2017-07-04

**Authors:** Tomoharu Shimizu, Toru Miyake, Masaji Tani

**Affiliations:** ^1^ Department of Surgery Shiga University of Medical Science Shiga Japan

**Keywords:** blood purification, direct hemoperfusion with Toraymyxin (PMX), endotoxin, sepsis, Toraymyxin

## Abstract

Toraymyxin^®^ (Toray Medical Co., Ltd, Tokyo, Japan) has been developed as a direct hemoperfusion column that contains polymyxin B‐immobilized fiber to bind endotoxins in patients’ blood. Toraymyxin was approved by the Japanese National Health Insurance system for the treatment of endotoxemia and septic shock in 1994. Since then, PMX (defined as direct hemoperfusion with Toraymyxin) has been safely used in more than 100 000 cases in emergency and intensive care units in Japan. Toraymyxin is currently available for use in clinical settings in 12 countries outside of Japan. We reviewed and analyzed the development, clinical use, and efficacy of Toraymyxin, and assessed the current status of Toraymyxin use for the treatment of severe sepsis and septic shock. Our review shows that PMX appeared to be effective in improving hemodynamics and respiratory function in septic shock requiring emergency abdominal surgery. Recent large‐scale ranomized controlled trialscould not demonstrate whether prognosis is improved by PMX. However, the latest meta‐analysis revealed that PMX significantly decreased mortality in patients with severe sepsis and septic shock. Combination of PMX with continuous hemodiafiltration and longer duration of PMX might be an effective strategy to improve survival in such patients.

## 1 INTRODUCTION

Despite recent advancements in surgical intervention and critical care, the prognosis of patients with endotoxemia and septic shock remains poor. Endotoxins are one of the major constituents of the cell walls of Gram‐negative bacteria. Endotoxins are recognized by Toll‐like receptor 4 that activates macrophages and other leukocytes to produce various inflammatory mediators. Endotoxins appeared to play a major role in the pathogenesis of Gram‐negative bacterial infections and in triggering toxic symptoms in patients with sepsis and septic shock. A recent review has shown that co‐detection of Gram‐negative bacteria and endotoxemia is predictive of an increased risk of mortality compared to the detection of neither.^1^ It is, therefore, reasonable to detect and remove circulating endotoxins in the blood of patients with Gram‐negative bacterial infections.

Polymyxin B is an antibiotic that displays strong bactericidal activity against Gram‐negative bacteria; it binds to and inactivates endotoxins.^2^ Giving systemic polymyxin B in humans is restricted because of its nephrotoxicity and neurotoxicity. Polymyxin B is considered a strong candidate as a ligand for extracorporeal selective adsorption of circulating endotoxins in the blood. Toraymyxin^®^ (Toray Medical Co., Ltd, Tokyo, Japan) has been developed as a direct hemoperfusion column that contains polymyxin B‐immobilized fiber to bind endotoxins in patients’ blood.

A narrative literature review was carried out using PubMed, MEDLINE and Google scholar search engines regarding PMX (defined as direct hemoperfusion with Toraymyxin). We describe the history of the development of Toraymyxin and comment on its clinical efficacy and current status with respect to its use for the treatment of severe sepsis and septic shock.

## 2 HISTORY OF THE DEVELOPMENT OF TORAYMYXIN

**Figure 1:**
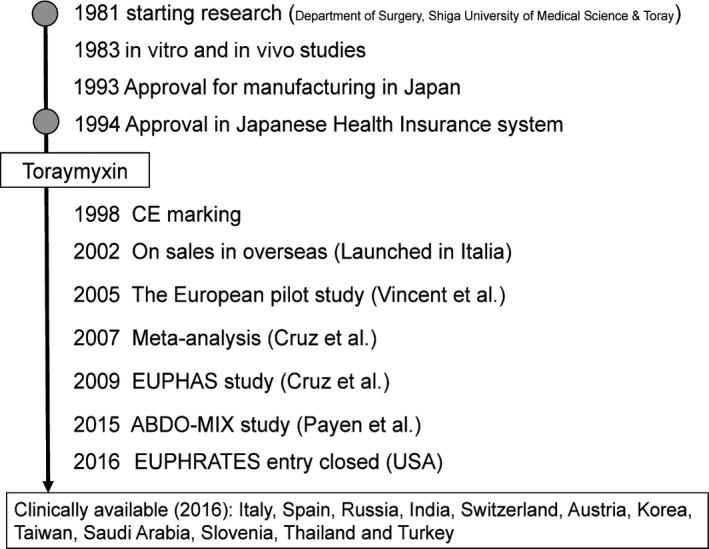
History of the development of Toraymyxin. EUPHAS, Early Use of Polymyxin B Hemoperfusion in Abdominal Sepsis. ABDO‐MIX trial, Effects of Hemoperfusion with a Polymyxin B Membrane in Peritonitis with Septic Shock. EUPHRATES trial, Evaluating the Use of Polymyxin B Hemoperfusion in a randomized controlled trial of Adults Treated for Endotoxemia and Septic Shock

Initial research into polymyxin B‐immobilized fibers began in 1981 at the Department of Surgery, Shiga University of Medical Science, Japan, as a collaboration between our research group and Toray Medical Co., Ltd (Figure 1).

A phase I clinical study was started in 1989, and the first clinical report of 16 patients with septic multiple organ failure treated with PMX was published in 1994.^3^ PMX was approved by the Japanese National Health Insurance system for the treatment of endotoxemia and septic shock in 1994.^4^ Since then, Toraymyxin has been safely used in more than 100 000 cases in emergency and intensive care units in Japan.

Toraymyxin received the CE mark of approval in Europe in 1998. Results of the first preliminary randomized controlled trial (RCT) in Europe were published in 2005, showing that treatment with PMX was safe and improves cardiac and renal dysfunction in patients with sepsis or septic shock.^5^ The findings of a meta‐analysis demonstrated the favorable effects of PMX in 2007.^6^ The results of a subsequent RCT in Europe, the EUPHAS study (Early Use of Polymyxin B Hemoperfusion in Abdominal Sepsis), which was conducted in Italy, were published in 2009, showing that PMX results in a significant reduction in sepsis‐associated mortality.^7^ A large RCT, the ABDO‐MIX trial (Effects of Hemoperfusion with a Polymyxin B Membrane in Peritonitis with Septic Shock) in France, failed to show a survival benefit and improvement in organ failure with PMX compared to the conventional treatment of peritonitis‐induced septic shock.^8^ Another large RCT, the EUPHRATES trial (Evaluating the Use of Polymyxin B Hemoperfusion in an RCT of Adults Treated for Endotoxemia and Septic Shock) in the USA and Canada,^9^ is closed to new patients and analysis is ongoing.

Toraymyxin is currently available for use in clinical settings in 12 countries outside of Japan (Italy, Spain, Russia, India, Switzerland, Austria, Korea, Taiwan, Saudi Arabia, Slovenia, Thailand, and Turkey) (Figure 1).

## 3 TORAYMYXIN (POLYMYXIN B‐IMMOBILIZED FIBER COLUMN)

**Figure 2:**
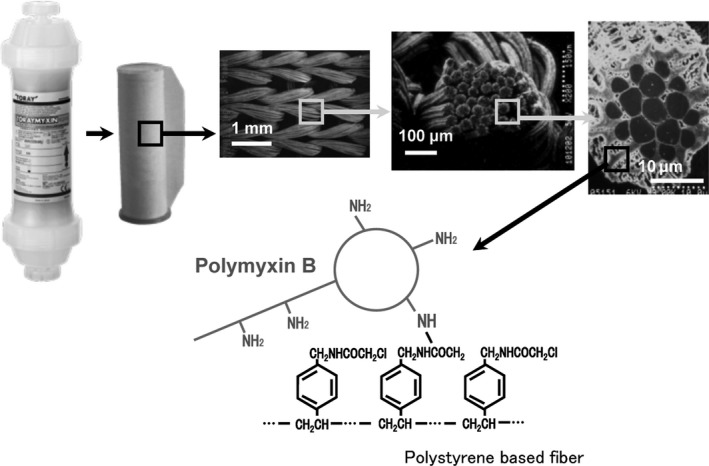
Physical structure of a Toraymyxin cartridge and of the knitted fabric roll of polymyxin B‐immobilized fibers (provided by Toray Medical Co., Ltd). The Toraymyxin cartridge contains a roll of knitted fibers. Each fiber consists of a bundle of ultrafine fibers with a diameter of approximately 20 μm. The polymyxin B molecules are covalently bound onto the fiber surface and therefore do not leak into the patient. Molecular conformation is shown. Polymyxin B is covalently bound to polystyrene‐based fiber

Toraymyxin comprises a plastic column containing a knitted roll of polymyxin B‐immobilized fiber fabric for human use. To enable the selective adsorption of circulating endotoxins in the blood, polymyxin B was covalently immobilized on the surface of polystyrene‐derived polypropylene‐reinforced conjugated carrier fibers (Figure 2).^10^

**Figure 3:**
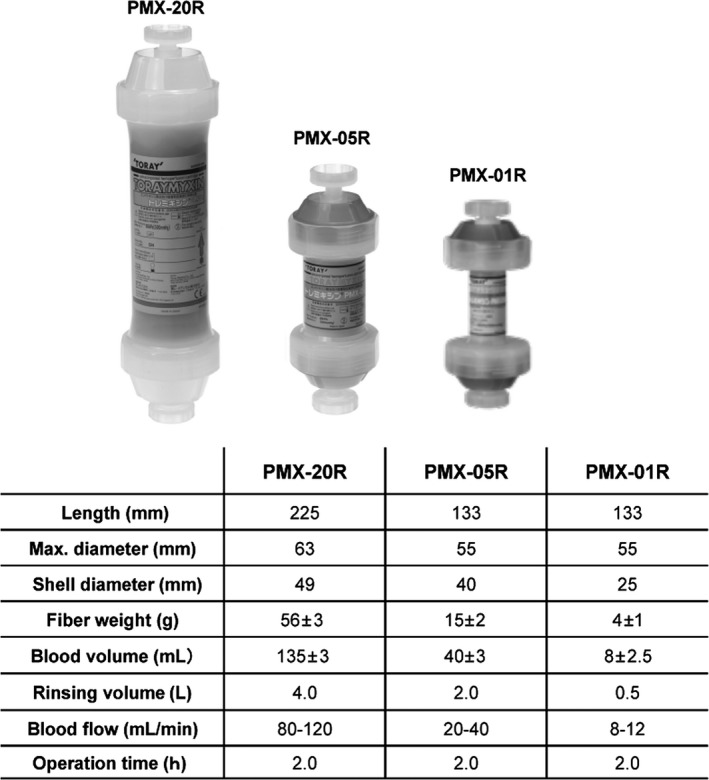
Specification of Toraymyxin cartridge. Overview of the three Toraymyxin cartridges currently available for clinical use (provided by Toray Medical Co., Ltd)

There are three types of Toraymyxin columns currently available for clinical use in Japan: PMX‐20R^®^ (volume, 135 mL), PMX‐05R^®^ (40 mL), and PMX‐01R^®^ (8 mL) (Figure 3). The first Toraymyxin column to be released was PMX‐20R, which was developed for the treatment of septic shock in adults.^4^ The second column to be released was PMX‐05R in 2005, which was developed for use in pediatric or elderly patients with smaller circulating blood volumes or bodyweight less than 40 kg.^11^ Several reports have shown acceptable results by using PMX‐05R in pediatric or elderly patients with sepsis.^11^ PMX‐01R was released for use in newborn or premature infants in 2011. There are a few case reports describing successful clinical experience with the use of PMX‐01R in patients with severe sepsis and a bodyweight less than 1000 g.^12^, ^13^

Since 1994, Toraymyxin has been used in over 100 000 cases in Japan. There have been no reports of any serious adverse effects. Despite adverse effects such as clotting of cartridges, thrombocytopenia or hypotension being reported, these incidents are rare.^4^

**Figure 4:**
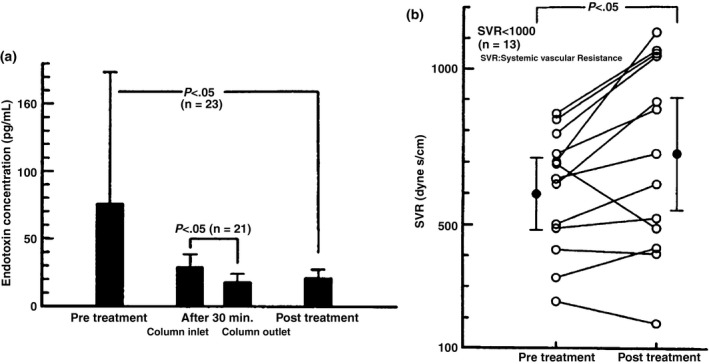
Results from the first clinical trial of Toraymyxin in Japan. (A) Endotoxin concentration before and after Toraymyxin treatment. (B) Systemic vascular resistance (SVR) before and after Toraymyxin treatment. Toraymyxin treatment decreased the concentration of endotoxin in the blood and improved hemodynamic status in patients with severe sepsis and septic shock. (Reproduced from Aoki et al. 1994.^3^)

The Toraymyxin column was originally designed to specifically adsorb endotoxins. Plasma endotoxin levels are significantly decreased immediately after PMX compared with pretreatment levels (Figure 4A). Furthermore, the patients’ hyperdynamic state, which is characterized by an increase in cardiac index during endotoxic shock, was returned to normal after treatment (Figure 4B).^3^ Almost all of the studies conducted to date have demonstrated a significant decrease in plasma endotoxin levels following PMX treatment. Although endotoxin‐specific Limulus amebocyte lysate (LAL) reagents can be used to accurately detect endotoxins, they are seldom used as a diagnostic tool in the clinical setting.^14^ Our previous study showed that sensitivity for detection of endotoxins of the turbidimetric LAL assay was very low (26.9%; 14 of 52 patients) in patients with severe sepsis and septic shock who required PMX treatment.^15^ A new method for the detection and quantitation of endotoxins is therefore urgently needed.

Recently, a new rapid assay called the endotoxin activity assay (EAA) had been developed, which detects endotoxins in whole blood by using autologous neutrophil‐dependent chemiluminescence.^16^ The EAA is currently approved by the United States Food and Drug Administration as a clinical method for the diagnosis of endotoxemia and has been used as one of the entry criteria in the EUPHRATES trial.^17^

## 4 CURRENT GUIDELINES IN THE CLINICAL USE OF PMX

The latest international guidelines, Surviving Sepsis Campaign: international guidelines for the management of severe sepsis and septic shock, 2012, was published in 2013.^18^ These guidelines are the third revised version of the international guidelines. PMX was not discussed in these guidelines because of the lack of sufficient evidence to improve the survival benefit of septic patients. It is still unclear whether PMX would be recommended in the next version of the international guidelines in light of the findings of these two recent large RCT.

The Japanese guidelines for the management of sepsis were published by the Japanese Society of Intensive Care Medicine in 2013.^19^ These guidelines discussed the clinical question of the efficacy of PMX in the treatment of septic shock and described that there were improvements in hemodynamics and respiratory function in patients with septic shock requiring emergency abdominal surgery; however, the evidence was insufficient to conclude whether the prognosis was improved by PMX treatment (weak recommendation).

The Third International Consensus Definitions for Sepsis and Septic Shock (Sepsis‐3) was published by the Society of Critical Care Medicine and the European Society of Intensive Care Medicine in 2016.^20^ The definitions of sepsis and septic shock have been extensively revised. Sepsis should be defined as life‐threatening organ dysfunction caused by a dysregulated host response to infection. For clinical operationalization, organ dysfunction can be represented by an increase in the Sequential Organ Failure Assessment (SOFA) score of two points or more.^20^ Patients with septic shock can be clinically identified by a vasopressor requirement to maintain a mean arterial pressure of 65 mm Hg or greater and a serum lactate level greater than 2 mmol/L (>18 mg/dL), in the absence of hypovolemia. Patients fulfilling the new definition of septic shock are likely to adapt to PMX; we have no evidence regarding the relation between Sepsis‐3 and PMX. Further studies are needed to clarify the relation of the new definition of sepsis and PMX.^20^

## 5 CLINICAL EVIDENCE FOR THE USE OF PMX IN THE TREATMENT OF SEVERE SEPSIS AND SEPTIC SHOCK

**Figure 5:**
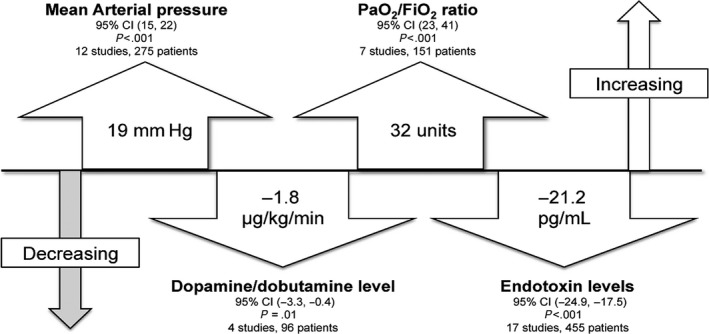
Main results of a meta‐analysis of Toraymyxin treatment. CI, confidence interval; MAP, mean arterial pressure; PaO_2_/FIO_2_, ratio of partial pressure arterial oxygen and fraction of inspired oxygen

Cruz et al.^6^ published a systematic review of the effectiveness of Toraymyxin for the treatment of sepsis in 2007. They included a total of 28 publications that reported at least one of the specified outcome measures for PMX. In this meta‐analysis of 1425 patients (PMX therapy, 978 patients; conventional therapy, 447 patients), PMX therapy was associated with a significantly lower risk of mortality compared with conventional therapy (PMX, 33.5% vs conventional treatment, 66.5%; risk ratio, 0.53; 95% confidence interval [CI], 0.43‐0.65). A 33% to 80% reduction in plasma endotoxin levels was also observed when compared to pretreatment levels. The large decrease in mortality was associated with an improvement in hemodynamic conditions after PMX; mean arterial pressure was significantly increased by 19 mmHg (mean increase, 26%; range, 14‐42%) and dopamine/dobutamine dose was decreased by 1.8 μg/kg per min. An improvement in pulmonary function was also demonstrated; mean ratio of partial pressure arterial oxygen to the fraction of inspired oxygen (PaO_2_/FiO_2_) was increased by 32 units (95% CI, 23‐41 units; *P*<.001) (Figure 5).

There are four RCT conducted outside of Japan as listed in Table 1. Vincent et al.^5^ published the results of a multicenter, open‐label, pilot, randomized controlled study conducted in intensive care units of six academic medical centers in Europe in 2005, which was the first RCT of Toraymyxin conducted outside of Japan. Thirty‐six postoperative patients with severe sepsis or septic shock secondary to intra‐abdominal infection were randomized to either PMX (n=17) or standard therapy (n=19), for 2 hours. PMX was well tolerated, and no significant adverse effects were observed. Patients treated with PMX showed significant increases in cardiac index (*P*=.012 and *P*=.032 at day 1 and day 2, respectively), left ventricular stroke work index (*P*=.015 at day 2), and oxygen delivery index (*P*=.007 at day 2) compared with controls. Furthermore, the need for continuous renal replacement therapy (CRRT) after study entry was significantly reduced in the PMX group (*P*=.043). There were no statistically significant differences in the change in endotoxin or interleukin (IL)‐6 levels, organ dysfunction as assessed using the SOFA scores, or 28‐day mortality. Together, these results showed that PMX treatment was safe and that it improved cardiac and renal dysfunction as a result of sepsis or septic shock.

**Table 1:** Summary of randomized controlled studies conducted outside of Japan

	European pilot study	EUPHAS	ABDO‐MIX	EUPHRATES
Trial method	Open‐label, pilot, RCT	Open‐label RCT	Open‐label RCT	Double blind, RCT
Country	Europe	Italy	France	USA, Canada
No. cases	36 (PMX 17, Control 19)	64 (PMX 34, Control 30)	243 (PMX 119, Control 113)	293 (PMX 144, Control 149)
Patients	Surgical patients with severe sepsis as a result of Gram‐negative abdominal infection	Severe sepsis or septic shock as a result of intra‐abdominal infection requiring emergency surgery	Septic shock as a result of peritonitis requiring emergency abdominal surgery	Septic shock EAA >0.6, MODS >9
PMX treatment	1 session of PMX 2 h	2 sessions of PMX 2 h, 24 h interval	2 sessions of PMX 2 h, 24 h interval	2 sessions of PMX 2 h, 24 h interval
Initiation of first PMX	Within 24 h (elective) or 48 h (emergency)	Within 24 h after surgery	Within 12 h after surgery	Within 24 h after EAA measurement
Primary endpoint	Improvement in organ dysfunction (SOFA score)	Baseline to 72 h in MAP and vasopressor requirement	28‐day mortality	28‐day mortality
Secondary endpoint	Plasma endotoxin and IL‐6, 28‐day mortality, length of ICU stay, hemodynamic data, need for RRT	PaO_2_/FiO_2_ ratio, change in organ dysfunction (measured by delta SOFA score), 28‐day mortality, need for RRT, length of ICU, hospital stay, all‐cause hospital mortality	7‐, 14‐, 21‐, and 90‐day mortality SOFA score variation within first 3days, time to withdraw catecholamine, adverse events	Survival time from baseline to death within 28‐day mortality, changes in organ dysfunction, MAP, CVI, renal function from baseline to day 3
28‐day mortality	PMX 29% (5/17) vs Conventional 28% (5/18)	PMX 32.4% (11/34) vs Conventional 53.3% (16/30)	PMX 27.7% (33/119) vs Conventional 19.5% (22/113)	PMX 43.75% (63/144) vs Control 44.3% (66/149)
Other results	No statistical significance in the change in endotoxin and IL‐6, organ dysfunction, significant improvement in cardiac and renal dysfunction	Significant improvement in MAP and inotropic score and vasopressor dependency index, PaO_2_/FiO_2_ ratio and SOFA score in PMX group	No statistical significance in SOFA score variation within first 3 days, time to withdraw catecholamine	Analysis is ongoing
Cartridge clotting	23.5% (4/17 sessions)	5.8% (4/68 sessions)	11.4% (25/220 sessions)	4.0% (17/424 sessions)
Year published	2005	2009	2015	R. P. Dellinger, unpublished preliminary report^26^
Reference number	5	7	8	9, 26

CVI, cardiovascular index; EEA, endotoxin activity assay; IL‐6, interleukin 6; MAP, mean arterial pressure; MODS, multiple organ dysfunction score; PMX, direct hemoperfusion with Toraymyxin; RCT, randomized controlled trial; RRT, renal replacement therapy; SOFA, Sequential Organ Failure Assessment.

Cruz et al.^7^ published the results of the EUPHAS trial, a prospective, multicenter, RCT conducted at the intensive care units of 10 Italian tertiary care hospitals in 2009. Sixty‐four patients with severe sepsis or septic shock who had undergone emergency surgery for intra‐abdominal infection were enrolled in this study. Patients were randomized within 6 hours after open abdominal surgery to either conventional therapy (n=30) or conventional therapy plus two sessions of 2‐h PMX with an interval of 24 hours between sessions (n=34). The 28‐day mortality was significantly improved in the PMX group (32%; 11/34 patients) compared with that in the conventional therapy group (53%; 16/30 patients). In the PMX group, mean arterial pressure significantly increased from 76 mmHg (before treatment) to 84 mmHg (72 hours after treatment; *P*=.001) and vasopressor requirement (measured as inotropic score) significantly decreased from 29.9 (before treatment) to 6.8 (72 hours after treatment; *P*<.001). In contrast, in the conventional therapy group, the mean arterial pressure (before, 74 mmHg; 72 hours after, 77 mmHg; *P*=.37) and inotropic score (before treatment, 28.6; after treatment, 22.4; *P*=.37) did not change significantly with treatment. PaO_2_/FiO_2_ ratio increased significantly (before treatment, 235; after treatment, 264; *P*=.049) in the PMX group but not in the conventional therapy group (before treatment, 217; after treatment, 228; *P*=.79). Moreover, the SOFA score, which is an indicator of the severity of organ dysfunction, was improved in the PMX group compared to the conventional therapy group (change in SOFA score, −3.4 vs −0.1; *P*<.001). Although the goal was to enrol 120 patients in this study, interim analysis revealed that the risk of mortality in the conventional treatment group was significantly higher than in the PMX group; thus, the EUPHAS study was terminated halfway. A Cox proportional hazard analysis was used for survival analysis in the EUPHAS study; however, some researchers questioned the analytical approach used in this study. Odds ratio (OR) of the crude 28‐day mortality rates was not statistically significant (11/34 vs 16/30; OR, 0.42; 95% CI, 0.13‐1.29; *P*=.13), using Fisher's exact test. Amaral^21^pointed out that a survival time analysis is inappropriate and potentially misleading in studies of critical illness outcomes with hospital censoring; so 28‐day, 60‐day, or hospital mortality might be a useful pilot trial endpoint in such studies. In an author reply, Antonelli and Ronco showed that the OR for hospital mortality was 0.35 (95% CI, 0.13‐0.97; *P*=.049), consistent with a statistically significant decrease in risk. They also showed that the difference of 60‐day mortality may not decrease with time.^21^ Despite the problem of statistical analysis on mortality, the high mortality rate in the conventional treatment group and the lack of evaluation of circulating endotoxin levels, this study demonstrated improvement in hemodynamic conditions in patients with severe sepsis and septic shock as primary endpoints of an RCT conducted outside of Japan.

The ABDO‐MIX trial was conducted in France.^8^ A total of 243 patients with peritonitis‐induced septic shock from abdominal infections were enrolled, and the primary endpoint of the study was the 28‐day mortality. No significant increase in mortality or improvement in organ failure in patients treated with PMX was observed compared to conventional therapy for peritonitis‐induced septic shock. However, major differences in the mortality rate and completion rate of two scheduled sessions of PMX among previous trials have since been reported.^22^ The 28‐day mortality rate recorded in both groups was significantly lower than that reported in larger studies (between 32.7 and 53% for similar patient cohorts).^23^, ^24^ Furthermore, only 81 of the 119 treated patients (68%) completed the two scheduled sessions of PMX. All patients enrolled in the previously published EUPHAS study had completed the two planned sessions of PMX with higher mortality observed in the control group.^22^ The incidence of cartridge clotting in the ABDO‐MIX study (11.4%; 25/220 sessions) was higher than in previous reports, such as 5.8% (4/68 sessions) in the EUPHAS study^7^ and 1.0% (12/1152 sessions) in the EUPHAS 2 study.^25^ Clotting problems in the blood circuit during PMX were seldom raised in Japan. The cause of death was also not clarified between the PMX and conventional groups in this study. As such, the conflicting results of the ABDO‐MIX study were inconclusive and not definitive.

The EUPHRATES trial conducted in the USA and Canada^9^ was designed to address the criticisms of previous studies. Circulating endotoxin levels in patients with septic shock were evaluated by means of EAA. A total of 293 patients with septic shock with high EAA activity (>0.6) and multiple organ dysfunction scores (MODS) (>9) were enrolled and randomized to either the PMX or conventional treatment groups. Dellinger showed a preliminary report at the 2016 annual meeting of the European Society of Intensive Care Medicine.^26^ The primary endpoint of mortality rate (44.3% in placebo group and 43.75% in PMX group) was not met in full intention‐to‐treat populations. Analyses of the secondary endpoints in this study are still ongoing and will be reported in the near future.

In their latest meta‐analysis, Terayama et al.^27^ recently demonstrated that PMX was associated with a lower mortality (risk ratio, 0.65; 95% CI, 0.47‐0.89; *P*=.007; I^2^=72%). In the subgroup analysis, they also showed that four studies published in Japan showed significantly lower mortality in the PMX group than in the control group (risk ratio, 0.53; 95% CI, 0.41‐0.69; *P*<.0001; I^2^=52%); however, three studies published in other countries failed to show a significant decrease in mortality in the PMX group (risk ratio, 0.98; 95% CI, 0.54‐1.78; *P*=.94; I^2^=60%). They discussed that a meta‐regression analysis revealed a significant negative slope between effect size of PMX therapy and baseline mortality rate in individual studies. Further, in this meta‐analysis, they suggested that a beneficial effect with PMX might be greater in patients with higher baseline mortality risk.^27^

The effect of postoperative PMX on mortality in patients with septic shock as a result of lower gastrointestinal tract perforation was examined by propensity‐matched analysis using the Japanese Diagnosis Procedure Combination (DPC) inpatient database.^28^ The 28‐day mortality in the PMX group (17.1%; 101/590) was not significantly different from that of the control group (16.3%; 96/590) (*P*=.696).^28^ The effect of PMX on mortality in patients with septic shock requiring CRRT was also examined using the DPC database, demonstrating that the 28‐day mortality in the PMX group (40.2%; 393/978) was significantly lower than that in the control group (46.8%; 458/978) (*P*=.003).^29^ Therefore, the mortality rate in the control group was lower (16.3%) in patients with lower gastrointestinal tract perforation than in patients requiring CRRT (46.8%). These large‐scale retrospective studies also seemed to support the fact that PMX might be effective in patients with a higher baseline mortality risk.

**Figure 6:**
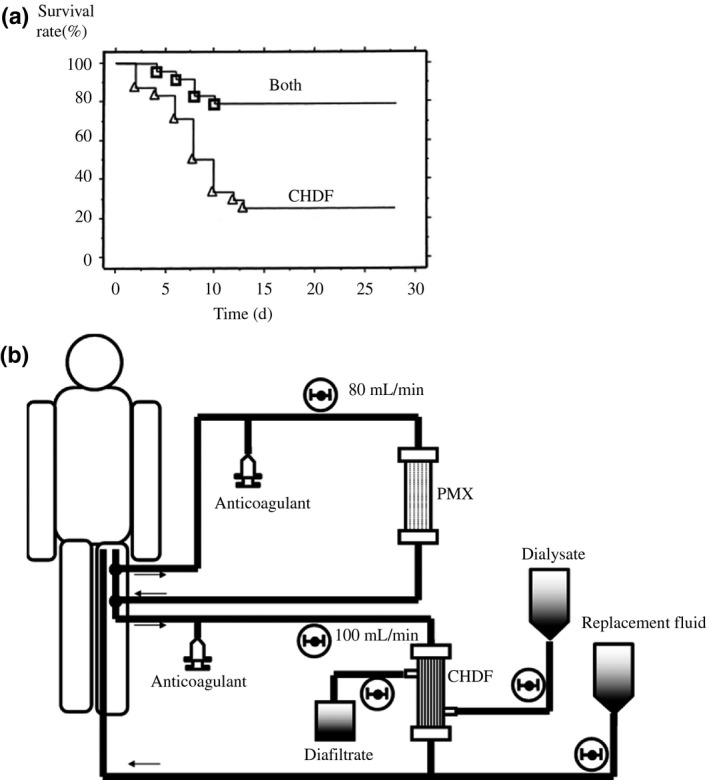
Combination use of Toraymyxin and continuous hemodiafiltration (CHDF). (A) Survival of patients receiving combination therapy of Toraymyxin and CHDF (Both). (Reproduced from Suzuki et al. 2002.^30^) Combination therapy of Toraymyxin and CHDF significantly improved survival rate in patients with sepsis and acute renal failure. (B) Schematic of the combination of PMX and CHDF in a series‐parallel circuit. (Reproduced from Yonekawa et al. 2006.^32^) PMX, direct hemoperfusion with Toraymyxin

The combination of PMX and continuous hemodiafiltration (CHDF) as CRRT appears to be more beneficial for patients with septic renal dysfunction than CHDF alone; combination treatment significantly decreases the concentration of circulating IL‐6 and improves patient survival (Figure 6A).^30^ Moreover, PMX followed by CHDF with a polymethyl methacrylate (PMMA) membrane significantly decreased the concentrations of plasminogen activator inhibitor‐1, protein C, interleukin‐6, and endogenous anandamide compared to PMX followed by CHDF with a polyacrylonitrile membrane in patients with septic shock.^31^ Therefore, the combination of PMX with PMMA‐CHDF is beneficial for patients with septic shock and septic acute kidney injury (AKI). In combination therapy, the Toraymyxin column is generally placed before or after CHDF on a single circuit. However, when CHDF cannot be interrupted, for example, in patients with severe renal failure, the Toraymyxin circuit can be connected in parallel or in series with the CHDF circuit (Figure 6B).^32^ The latest meta‐analysis 27 and large‐scale retrospective studies 28, 29 suggested that a beneficial effect with PMX might be higher in patients with a higher baseline mortality risk; such patients required the combination of PMX with CHDF. Further studies are needed to clarify the proper indication and clinical benefits for the combination of PMX and CHDF in patients with septic shock.

Originally, the treatment duration of PMX was set at 2 hours following the results seen in preclinical studies (Figure 3). Endotoxin adsorption capacity is considered to reach equilibrium at 2 hours after the initiation of perfusion; however, the evident clinical efficacy of longer duration of PMX (>6 hours) has been reported in cases lacking clinical efficacy within 2 hours.^33^, ^34^, ^35^ Mitaka et al.^33^ have compared clinical data for patients with a longer duration of PMX (16.9±7.0 hours) with data for patients with the standard 2‐h duration. Yamashita et al.^35^ recently showed that a longer duration of PMX might improve the hemodynamics and pulmonary oxygenation capacity in patients without a favorable response to the 2‐h PMX. Adverse effects such as thrombocytopenia have not been reported despite a longer duration of PMX.^33^, ^34^ The four RCT listed in Table 1 used the standard 2‐h‐duration PMX. Further studies to investigate the survival benefits of long‐duration PMX treatment are expected in the future.

Source of the infection that induces septic shock is important in evaluating a patient's prognosis. The three RCT (European pilot study, EUPHAS, and ABDO‐MIX) and almost all studies published in Japan included patients with septic shock as a result of abdominal infections. The EUPHAS 2 registry was conducted by 57 centers collecting retrospective data for 357 patients with severe sepsis and septic shock.^25^ The respiratory source (17.6%; 63 cases) was, in fact, the second most frequently observed clinical condition in the study. The 28‐day mortality in patients with respiratory infections (52.5%) showed a trend toward a higher mortality rate than in those with abdominal infections (39.4%). Lower respiratory tract infections may have some differences compared to abdominal or urinary sepsis. In the discussion, they described that pulmonary sepsis seemed to respond less to the two sessions of endotoxin removal because source control is better achieved through antibiotic action and the resolution needs a longer time. However, in patients with intra‐abdominal sepsis, the source control is obtained through surgery and concomitant PMX treatment, achieving a more rapid resolution.^25^ Kono et al.^36^ reported that a longer duration of PMX is more efficacious for patients with acute exacerbation of interstitial pneumonia than a short duration. The longer duration of PMX might also improve the conditions of such patients with septic shock as a result of pulmonary infections.

## 6 CONCLUSION

PMX appeared to be effective in hemodynamics and respiratory function in patients with septic shock requiring emergency abdominal surgery. The recent large‐scale RCT could not demonstrate whether the prognosis is improved by PMX; however, the recent meta‐analysis revealed that PMX therapy significantly decreased mortality in patients with severe sepsis and septic shock. The combination of PMX with CHDF and longer duration of PMX might be an effective strategy to improve the survival of patients with severe sepsis and septic shock.

## DISCLOSURE

Conflict of Interest: Authors declare no conflicts of interest for this article.

## References

[1] Hurley JC , Guidet B , Offenstadt G , Maury E . Endotoxemia and mortality prediction in ICU and other settings: underlying risk and co‐detection of gram negative bacteremia are confounders. Crit Care. 2012;16:R148. 22871090 10.1186/cc11462 PMC3580737

[2] Anspach FB . Endotoxin removal by affinity sorbents. J Biochem Biophys Methods. 2001;49:665–81. 11694310 10.1016/s0165-022x(01)00228-7

[3] Aoki H , Kodama M , Tani T , Hanasawa K . Treatment of sepsis by extracorporeal elimination of endotoxin using polymyxin b‐immobilized fiber. Am J Surg. 1994;167:412–7. 8179086 10.1016/0002-9610(94)90126-0

[4] Shoji H . Extracorporeal endotoxin removal for the treatment of sepsis: endotoxin adsorption cartridge (toraymyxin). Ther Apher Dial. 2003;7:108–14. 12921125 10.1046/j.1526-0968.2003.00005.x

[5] Vincent JL , Laterre PF , Cohen J , et al. A pilot‐controlled study of a polymyxin b‐immobilized hemoperfusion cartridge in patients with severe sepsis secondary to intra‐abdominal infection. Shock. 2005;23:400–5. 15834304 10.1097/01.shk.0000159930.87737.8a

[6] Cruz DN , Perazella MA , Bellomo R , et al. Effectiveness of polymyxin b‐immobilized fiber column in sepsis: a systematic review. Crit Care. 2007;11:R47. 17448226 10.1186/cc5780 PMC2206475

[7] Cruz DN , Antonelli M , Fumagalli R , et al. Early use of polymyxin b hemoperfusion in abdominal septic shock: the EUPHAS randomized controlled trial. JAMA. 2009;301:2445–52. 19531784 10.1001/jama.2009.856

[8] Payen DM , Guilhot J , Launey Y , et al. Early use of polymyxin b hemoperfusion in patients with septic shock due to peritonitis: a multicenter randomized control trial. Intensive Care Med. 2015;41:975–84. 25862039 10.1007/s00134-015-3751-z PMC4477725

[9] Klein DJ , Foster D , Schorr CA , Kazempour K , Walker PM , Dellinger RP . The Euphrates trial (evaluating the use of polymyxin b hemoperfusion in a randomized controlled trial of adults treated for endotoxemia and septic shock): study protocol for a randomized controlled trial. Trials. 2014;15:218. 24916483 10.1186/1745-6215-15-218 PMC4066268

[10] Shoji H , Tani T , Hanasawa K , Kodama M . Extracorporeal endotoxin removal by polymyxin b immobilized fiber cartridge: designing and antiendotoxin efficacy in the clinical application. Ther Apher. 1998;2:3–12. 10227782 10.1111/j.1744-9987.1998.tb00066.x

[11] Nakamura T , Kawagoe Y , Suzuki T , Shoji H , Ueda Y , Koide H . Polymyxin b‐immobilized fiber hemoperfusion with the PMX‐05R column in elderly patients suffering from septic shock. Am J Med Sci. 2007;334:244–7. 18030179 10.1097/MAJ.0b013e3180a5e8d8

[12] Nishizaki N , Nakagawa M , Hara S , et al. Effect of PMX‐DHP for sepsis due to ESBL‐producing E. coli in an extremely low‐birthweight infant. Pediatr Int. 2016;58:411–4. 26710929 10.1111/ped.12825

[13] Tokumasu H , Watabe S , Tokumasu S . Effect of hemodiafiltration therapy in a low‐birthweight infant with congenital sepsis. Pediatr Int. 2016;58:237–40. 26669790 10.1111/ped.12776

[14] Venet C , Zeni F , Viallon A , et al. Endotoxaemia in patients with severe sepsis or septic shock. Intensive Care Med. 2000;26:538–44. 10923727 10.1007/s001340051201

[15] Shimizu T , Hanasawa K , Sato K , et al. Direct hemoperfusion with polymyxin‐b‐immobilized fiber columns improves septic hypotension and reduces inflammatory mediators in septic patients with colorectal perforation. Langenbecks Arch Surg. 2009;394:303–11. 18685861 10.1007/s00423-008-0395-2

[16] Romaschin AD , Harris DM , Ribeiro MB , et al. A rapid assay of endotoxin in whole blood using autologous neutrophil dependent chemiluminescence. J Immunol Methods. 1998;212:169–85. 9672205 10.1016/s0022-1759(98)00003-9

[17] Foster D , Klein DJ , Guadagni G , Walker PM . Endotoxin removal: bringing the mission to North America. Blood Purif. 2014;37(Suppl 1):14–7. 10.1159/000356832 24457490

[18] Dellinger RP , Levy MM , Rhodes A , et al. Surviving sepsis campaign: international guidelines for management of severe sepsis and septic shock, 2012. Intensive Care Med. 2013;39:165–228. 23361625 10.1007/s00134-012-2769-8 PMC7095153

[19] Oda S , Aibiki M , Ikeda T , et al. The Japanese guidelines for the management of sepsis. J Intensive Care. 2014;2:55. 25705413 10.1186/s40560-014-0055-2 PMC4336273

[20] Singer M , Deutschman CS , Seymour CW , et al. The third international consensus definitions for sepsis and septic shock (Sepsis‐3). JAMA. 2016;315:801–10. 26903338 10.1001/jama.2016.0287 PMC4968574

[21] Amaral AC . Polymyxin b hemoperfusion and mortality in abdominal septic shock. JAMA. 2009;302:1968–9; author reply 9‐70. 10.1001/jama.2009.1607 19903914

[22] Antonelli M , Ronco C . Polymyxin b hemoperfusion in sepsis: growing body of evidence and occasional conflicting results. Blood Purif. 2015;39:I–II. 10.1159/000431018 25998615

[23] Antonelli M , Fumagalli R , Cruz DN , Brienza N , Giunta F , Group ES . PMX endotoxin removal in the clinical practice: results from the EUPHAS trial. Contrib Nephrol. 2010;167:83–90. 20519902 10.1159/000315922

[24] Early Use of Polymyxin BHitASCG . Polymyxin b hemoperfusion in clinical practice: the picture from an unbound collaborative registry. Blood Purif. 2014;37(Suppl 1):22–5. 24457492 10.1159/000356835

[25] Cutuli SL , Artigas A , Fumagalli R , et al. Polymyxin‐b hemoperfusion in septic patients: analysis of a multicenter registry. Ann Intensive Care. 2016;6:77. 27502196 10.1186/s13613-016-0178-9 PMC4977232

[26] Dellinger RP . Evaluating the use of polymyxin b hemoperfusion in a RCT of adults treated for endotoxemia and septic shock. Annual meeting 2016 of European Society of Intensive Care Medicine. 2016 [cited 23 Jan 2017]. Available from: http://www.esicm.org/news-article/hot-topics-full-presentations.

[27] Terayama T , Yamakawa K , Umemura Y , Aihara M , Fujimi S . Polymyxin b hemoperfusion for sepsis and septic shock: a systematic review and meta‐analysis. Surg Infect (Larchmt). 2017;18:225–33. 28092497 10.1089/sur.2016.168

[28] Iwagami M , Yasunaga H , Doi K , et al. Postoperative polymyxin b hemoperfusion and mortality in patients with abdominal septic shock: a propensity‐matched analysis. Crit Care Med. 2014;42:1187–93. 24365858 10.1097/CCM.0000000000000150

[29] Iwagami M , Yasunaga H , Noiri E , et al. Potential survival benefit of polymyxin b hemoperfusion in septic shock patients on continuous renal replacement therapy: a propensity‐matched analysis. Blood Purif. 2016;42:9–17. 26918904 10.1159/000444474

[30] Suzuki H , Nemoto H , Nakamoto H , et al. Continuous hemodiafiltration with polymyxin‐b immobilized fiber is effective in patients with sepsis syndrome and acute renal failure. Ther Apher. 2002;6:234–40. 12109950 10.1046/j.1526-0968.2002.00416.x

[31] Sakamoto Y , Mashiko K , Obata T , et al. Effectiveness of continuous hemodiafiltration using a polymethylmethacrylate membrane hemofilter after polymyxin b‐immobilized fiber column therapy of septic shock. ASAIO J. 2008;54:129–32. 18204329 10.1097/MAT.0b013e31815d2f01

[32] Yonekawa C , Nakae H , Tajimi K , Asanuma Y . Combining continuous endotoxin apheresis and continuous hemodiafiltration in the treatment of patients with septic multiple organ dysfunction syndrome. Ther Apher Dial. 2006;10:19–24. 16556132 10.1111/j.1744-9987.2006.00341.x

[33] Mitaka C , Tsuchida N , Kawada K , Nakajima Y , Imai T , Sasaki S . A longer duration of polymyxin b‐immobilized fiber column hemoperfusion improves pulmonary oxygenation in patients with septic shock. Shock. 2009;32:478–83. 19295483 10.1097/SHK.0b013e3181a2a978

[34] Shimizu T , Obata T , Sonoda H , et al. The ability of endotoxin adsorption during a longer duration of direct hemoperfusion with a polymyxin b‐immobilized fiber column in patients with septic shock. Transfus Apher Sci. 2013;49:499–503. 23683501 10.1016/j.transci.2013.04.042

[35] Yamashita C , Hara Y , Kuriyama N , Nakamura T , Nishida O . Clinical effects of a longer duration of polymyxin b‐immobilized fiber column direct hemoperfusion therapy for severe sepsis and septic shock. Ther Apher Dial. 2015;19:316–23. 26386218 10.1111/1744-9987.12339

[36] Kono M , Suda T , Enomoto N , et al. Evaluation of different perfusion durations in direct hemoperfusion with polymyxin b‐immobilized fiber column therapy for acute exacerbation of interstitial pneumonias. Blood Purif. 2011;32:75–81. 21372564 10.1159/000320128

